# Proteomic profiling of epicardial fat in heart failure with preserved versus reduced and mildly reduced ejection fraction

**DOI:** 10.1111/jcmm.17695

**Published:** 2023-02-20

**Authors:** Qian Gao, Shan He, Yuanshu Peng, Pixiong Su, Lei Zhao

**Affiliations:** ^1^ Emergency Department, Beijing Shijitan Hospital Capital Medical University Beijing China; ^2^ Heart Center, Beijing Chaoyang Hospital Jingxi Branch Capital Medical University Beijing China; ^3^ Heart Center & Beijing Key Laboratory of Hypertension, Beijing Chaoyang Hospital Capital Medical University Beijing China

**Keywords:** epicardial adipose tissue, heart failure with mildly reduced ejection fraction, heart failure with preserved ejection fraction, heart failure with reduced ejection fraction, proteomics

## Abstract

In order to explore the proteomic signatures of epicardial adipose tissue (EAT) related to the mechanism of heart failure with reduced and mildly reduced ejection fraction (HFrEF/HFmrEF) and heart failure (HF) with preserved ejection fraction (HFpEF), a comprehensive proteomic analysis of EAT was made in HFrEF/HFmrEF (*n* = 5) and HFpEF (*n* = 5) patients with liquid chromatography–tandem mass spectrometry experiments. The selected differential proteins were verified between HFrEF/HFmrEF (*n* = 20) and HFpEF (*n* = 40) by ELISA (enzyme‐linked immunosorbent assay). A total of 599 EAT proteins were significantly different in expression between HFrEF/HFmrEF and HFpEF. Among the 599 proteins, 58 proteins increased in HFrEF/HFmrEF compared to HFpEF, whereas 541 proteins decreased in HFrEF/HFmrEF. Of these proteins, TGM2 in EAT was down‐regulated in HFrEF/HFmrEF patients and was confirmed to decrease in circulating plasma of the HFrEF/HFmrEF group (*p* = 0.019). Multivariate logistic regression analysis confirmed plasma TGM2 could be an independent predictor of HFrEF/HFmrEF (*p* = 0.033). Receiver operating curve analysis indicated that the combination of TGM2 and Gensini score improved the diagnostic value of HFrEF/HFmrEF (*p* = 0.002). In summary, for the first time, we described the proteome in EAT in both HFpEF and HFrEF/HFmrEF and identified a comprehensive dimension of potential targets for the mechanism behind the EF spectrum. Exploring the role of EAT may offer potential targets for preventive intervention of HF.

## 1 INTRODUCTION

Heart failure (HF) is a major healthcare, social and economic issue worldwide.^1^ Due to increasing ageing population and rising metabolic disorders, it has become a main cause of hospitalization. Based on left ventricular ejection fraction (LVEF), HF can be classified into ‘systolic’ HF with reduced or mildly reduced EF (HFrEF/ HFmrEF) and ‘diastolic’ HF with preserved EF (HFpEF), among which, HFpEF has become the most common form, constituting about 50% of the overall HF population,^2^ especially as seen in those elderly patients with comorbidities such as obesity, hypertension, diabetes and metabolic syndrome. The underlying pathophysiology by which these comorbidities contribute to cardiac function is not yet fully understood but is clearly related to metabolic duress and low‐grade inflammation.^2^, ^3^, ^4^ A growing body of evidence have also demonstrated that patients with HF are suffering lipid‐metabolic derangements,^5^, ^6^ substantially impacting long‐term outcome. Recently, adipose tissue of particular interest in the major risk factor of HF, especially HFpEF, is epicardial adipose tissue (EAT), since these fat deposits lie directly over the surface of the myocardium with no fascia separating the two issues.

EAT is located between the visceral pericardium and the outer myocardium, and they share the same microvasculature. Depending on different physiological conditions, EAT acts as a buffer to absorb fatty acids or take pathologic metabolic activities after a ‘phenotypic’ transformation.^5^, ^7^ The relationship between increased EAT volume and HF has already been established. As previously described, increased EAT correlated with incident HF in the general population, especially HFpEF,^8^, ^9^ while a single study suggested EAT was reduced in patients with HFrEF.^10^ Recently, a study by Pugliese et al.^11^ posed an intriguing observation that indicated the role of EAT on HF might be divergent, in which, for HFpEF, EAT accumulation is related to worse haemodynamic and metabolic profiles, whereas in HFrEF, less EAT suggested more severe cardiac function and adverse prognosis. The results of Pugliese provided compelling clinical evidence on the diverging role of EAT across the EF spectrum, but the molecular signatures behind the pathophysiological of EAT on HFrEF/HFmrEF and HFpEF have not been systematically explored. Besides, Jin et al.^12^ reconfirmed a greater thickness EAT in patients with HFpEF than HFrEF/HFmrEF, and increased EAT thickness is associated with worse left atrial and ventricular function in HFpEF but opposite in HFrEF/HFmrEF. Our previous study^5^ has pictured a comprehensive profile for connecting EAT with the pathogenesis of HF. Nevertheless, the proteomic difference of EAT between HFrEF/HFmrEF and HFpEF remains largely unknown. Accordingly, in the present study, we intend to take a comprehensive proteomic analysis of EAT in patients with HFrEF/HFmrEF and HFpEF and investigate the substrate molecular changes that may be involved in this pathology.

## 2 METHODS

### 2.1 Study population and tissue sample

EAT were taken from 10 patients who were diagnosed with HF undergoing coronary artery bypass grafting or cardiac surgery for valve replacement between May 2021 and August 2021 and were divided into HFrEF/HFmrEF (*n* = 5) or HFpEF (*n* = 5) groups. The clinical manifestation and examinations [brain natriuretic peptide (BNP) >500 ng/L, enlarged left ventricular end‐diastolic diameter and reduced left ventricular ejection fraction (<50%)] were the criteria for diagnosing HF. HFrEF/HFmrEF was defined by an LVEF <50%, while HFpEF required an LVEF ≥50%, N‐terminal pro‐B‐type natriuretic peptide (NT‐proBNP) >125 pg/mL and the additional presence of relevant structural heart disease or diastolic dysfunction.^13^ Gensini Score which was a degree of stenosis score was multiplied by the lesion site score, and the sum of the lesion score was taken as the final score.^14^ Baseline demographic characteristics and clinical data were taken at admission. Approximately 2 cm^3^ EAT samples were taken from the left‐interventricular groove, cut into small pieces, washed three times with ice‐cold phosphate buffer saline, frozen for 10 min in liquid nitrogen and then stored at −80°C until analysis. All samples were collected a few minutes before the extracorporeal circulation under the same haemodynamic conditions. All procedures followed were in accordance with the ethical standards of the responsible committee on human experimentation (the Ethical Committee of Beijing Chaoyang Hospital, China).

### 2.2 Extraction of protein from epicardial adipose tissue

Proteins of EAT were extracted using RadioImmuno Precipitation Assay buffer (Solarbio, Beijing) and protease inhibitors (Sigma‐Aldrich, St. Louis). BCA protein assay (Pierce, IL) was used to measure the protein concentration. Pre‐chilled acetone was added to 100‐μg alkylated proteins. Extracted proteins were solubilized in ammonium bicarbonate (100 mmol/L) by adding sodium deoxycholate (SDC) (1%) and reduced with tris 2‐carboxyethyl phosphine hydrochloride (5 mmol/L) for 10 min at 55°C, followed by alkylation with iodoacetamide (10 mmol/L) for 15 min at room temperature in the dark. And then digested at 37°C overnight with sequencing grade modified trypsin (trypsin 1:50 protein w/w ratio; Promega, Madison, WI). The tryptic peptides were desalted using a C18 cartridge (3 M, St. Paul) after cleaning up SDC with TFA (2%) and then dried in a vacuum concentrator.

### 2.3 Label‐free quantitative proteomics by LC–MS/MS

Extracted peptides were separated at a 300 nL/min flow rate using a reversed‐phase column (100 μm × 150 mm, 3 μm ReproSil‐Pur 120 C18‐AQ, 1.9 μm, Dr Math), followed by analysing with the EASY‐nLC nano‐UPLC (Thermo Fisher Scientific, Bremen, Germany). It performed at a 120‐min gradient elution with the UPLC mobile phase A [formic acid (FA) (0.1%) with acetonitrile (ACN) (2%)] and B [ACN (80%) with FA (0.1%)] (8%–30% B for 92 min, 30%–40% B for 20 min, 40%–100% B for 2 min, 100% B for 2 min, 100%–2% B for 2 min and finally 2% B for 2 min). Q‐Exactive mass spectrometer (Thermo Fisher Scientific, Bremen, Germany) applying the DDA (data‐dependent acquisition) strategy which MS for the 20 most intense ions using a normalized collision energy of 27% for HCD and an isolation window of 2 m/z were used to analyse the peptides. Resolution of 70,000 for MS1 (at 200 m/z) and 17,500 for MS2 in an orbitrap analyser were processed in the following analyses. The automatic gain control target for MS1 and MS2 were set to 3.0 E^+6^ with max IT 50 ms and 5.0 E^+4^ with max IT 100 ms respectively. Thirty seconds were set for dynamic exclusion.

### 2.4 Bioinformatics analysis

MaxQuant software (version 1.5.6.0) was used to perform the raw data. The following parameters were carbamidomethyl [C] as fixed modification, oxidation [M] and acetyl [protein N‐term] as variable modifications, three missed cleavages were allowed. The false discovery rates (FDRs) of the peptide‐spectra matches were set to less than 0.01 which were determined by a decoy database (Uniprot_human_2016_09). Only unique and razor peptides were quantified. All the others were reserved as default. Proteins established more than 99% probability with obtained two correct assigned peptides were considered to be identified. Normalized spectral protein intensity (LFQ intensity) was used to calculate the protein abundance. Perseus program was used to calculate the significance of Log_2_LFQ intensity of proteins between HFrEF/HFmrEF and HFpEF groups. The analysis of intracellular pathways was performed by searching Gene Ontology (GO) and Kyoto Encyclopedia of Genes and Genomes (KEGG) database.

### 2.5 Measurement of TGM2 levels in plasma

Peripheral venous blood was collected in a tube containing potassium EDTA and was centrifuged at 1500 *g* for 10 min at ambient temperature immediately. Plasma from the supernatant was collected and frozen at −80°C until analysis. Plasma samples were diluted 1:50000 when tested. Plasma concentrations of TGM2 (Protein‐glutamine gamma‐glutamyltransferase 2) were measured by ELISA (enzyme‐linked immunosorbent assay) from a commercially available microplate kit (CUSABIO Biotech Co. Ltd) in accordance with the manufacturer's instructions. The intra‐ and inter‐assay coefficients of variation were both less than 5%.

### 2.6 Statistics

Continuous variables were expressed as the mean ± standard deviation for normal distribution or median (interquartile range) for non‐normal distribution. Normality was processed using the Kolmogorov–Smirnov test. Categorical variables were expressed as frequencies and percentages. Comparisons between groups were performed using independent‐sample *t* test or Mann–Whitney U tests for continuous variables and Chi‐square or Fisher's exact tests for categorical variables. Binary logistic regression was used to identify the variables associated with HFrEF/HFmrEF in patients with HF. A receiver operator curve (ROC) and the area under the curve (AUC) for the prediction of HFrEF/HFmrEF and HFpEF were analysed. A two‐tailed value of *p* < 0.05 was considered statistically significant with SPSS software (version 23.0, IBM Corp, Armonk, NY).

## 3 RESULTS

### 3.1 Patient characteristics

Initially, a total of 10 HF patients were enrolled and were divided into HFrEF/HFmrEF or HFpEF groups. The demographic and clinical parameters of the patients are illustrated in Table 1. EF (42.7 ± 9.65 vs 61.8 ± 4.92) and LV end‐diastolic dimension (60.4 ± 5.59 vs 50.0 ± 5.43) were significantly different between HFrEF/HFmrEF and HFpEF groups among all parameters (*p* = 0.004, 0.018, respectively).

**TABLE 1:** Baseline characteristics of heart failure patients.

	HFrEF/HFmrEF (*n* = 5)	HFpEF (*n* = 5)	*p* value
Age (years)	67.8 ± 4.87	58.4 ± 10.14	0.099
Male, *n* (%)	3 (60%)	3 (60%)	/
BMI	23.87 ± 4.56	25.52 ± 5.15	0.604
BSA (m^2^)	1.67 ± 0.16	1.73 ± 0.19	0.575
FBG (mmol/L)	11.76 ± 6.41	4.79 ± 0.28	0.072
CHO (mmol/L)	3.67 ± 3.07	4.43 ± 1.04	0.625
HDL (mmol/L)	0.84 ± 0.54	1.16 ± 0.35	0.298
LDL (mmol/L)	2.34 ± 2.52	2.48 ± 0.90	0.912
TG (mmol/L)	0.89 ± 0.38	1.34 ± 0.53	0.164
UA (U/L)	363.2 ± 82.37	336.3 ± 62.92	0.578
Cr (umol/L)	88.2 ± 25.22	70.14 ± 23.8	0.277
CRP (mg/L)	6.51 ± 6.73	3.12 ± 2.84	0.401
BNP (pg/L)	1935.4 ± 1745.26	457.8 ± 557.11	0.109
EF (%)	42.7 ± 9.65	61.8 ± 4.92	0.004**
LVEDD (mm)	60.4 ± 5.59	50.0 ± 5.43	0.018*

*Note*: Data are mean ± SD or number (%). *P < 0.05, **P < 0.01.

Abbreviations: BMI, body mass index; BNP, brain natriuretic peptide; BSA, body surface area; CHO, cholesterol; Cr, creatinine; CRP, C reactive protein; EF, ejection fraction; FBG, fasting blood glucose; HDL, high‐density lipoprotein; LDL, low‐density lipoprotein; LVEDD, left ventricular end‐diastolic dimension; TG, triglyceride; UA, uric acid.

### 3.2 Human EAT proteome profile between HFrEF/HFmrEF and HFpEF group

**FIGURE 1:**
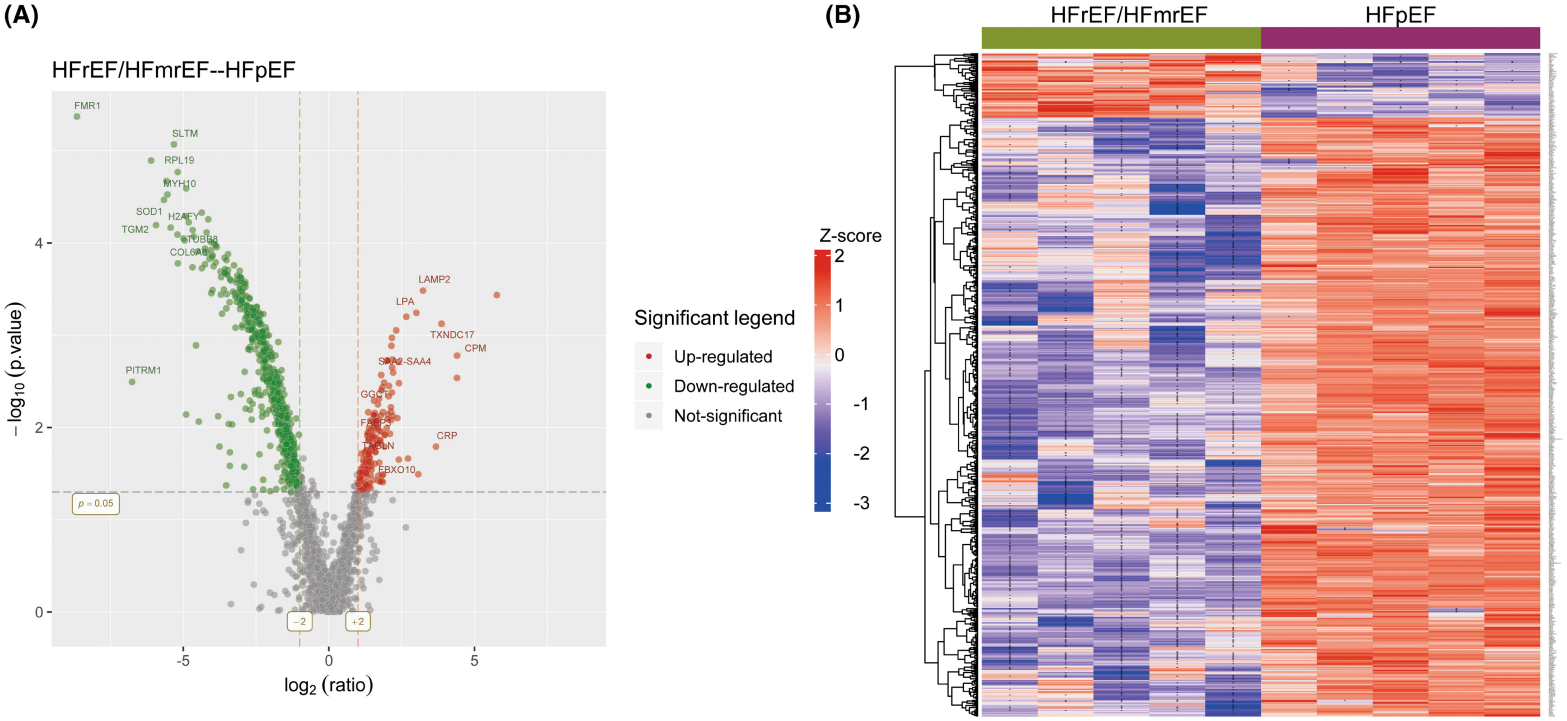
Comprehensive proteome profile of from epicardial adipose 10 patients. (A) Volcano/foldchange plot described significant proteins tissue in HFrEF/HFmrEF and HFpEF groups. The red dots represent up‐regulated and the green dots represent the down‐regulated. (B) Cluster heat map of sample performed in two groups by hierarchical clustering [HFrEF/HFmrEF (*n* = 5) and HFpEF (*n* = 5)].

We got a core set of 2407 quantified proteins in HFrEF/HFmrEF and HFpEF groups by importing the raw data to LC–MS/MS datHFrEF/HFmrEF and HFpEF abase and searching it. Of these, 599 EAT proteins were significantly differentially expressed between the two groups. Of those 599 proteins, 58 proteins increased in HFrEF/HFmrEF relative to HFpEF, whereas 541 proteins decreased in HFrEF/HFmrEF. The volcano plots (Figure 1A) showed the significant distribution and variation range of differential proteins between the HFrEF/HFmrEF and HFpEF group. The cluster heat map (Figure 1B), separated groups with the EF in HF patients, using hierarchical analysis with a Pearson correlation. Based on the protein ratios of the 599 significant expressed EAT proteins, we identified the 10 most significantly up and down expressed proteins between the HFrEF/HFmrEF and HFpEF group (Table 2). Of these, TGM2 was significantly down‐regulated in the HFrEF/HFmrEF group (*p* < 0.001).

**TABLE 2:** Ten significantly up‐ and down‐expression proteins in EAT identified by LC–MS/MS (HFrEF/HFmrEF vs. HFpEF) (*p* < 0.05).

Protein name	UniProt ID	Number of unique peptides	% sequence coverage	*p* value	Foldchange (HFrEF/HFmrEF:HFpEF)
Synaptic functional regulator	FMR1_HUMAN	2	2.8%	<0.001	0.002
Presequence protease, mitochondrial	PREP_HUMAN	2	2%	0.003	0.009
Protein‐glutamine gamma‐glutamyltransferase 2	TGM2_HUMAN	29	41.3%	<0.001	0.016
Superoxide dismutase	SODC_HUMAN	10	84.4%	<0.001	0.019
Myosin‐10	MYH10_HUMAN	54	40.6%	<0.001	0.021
Core histone macro‐H2A.1	H2AY_HUMAN	11	40.7%	<0.001	0.023
SAFB‐like transcription modulator	SLTM_HUMAN	2	46.8%	<0.001	0.025
Tubulin beta‐8 chain	TBB8_HUMAN	2	24.5%	<0.001	0.027
60S ribosomal protein L19	RL19_HUMAN	3	18.7%	<0.001	0.027
Collagen alpha‐6 (VI) chain	CO6A6_HUMAN	39	21.9%	<0.001	0.027
Carboxypeptidase M	CBPM_HUMAN	3	6.1%	0.002	21.120
Thioredoxin domain‐containing protein 17	TXD17_HUMAN	3	30.1%	<0.001	14.601
C‐reactive protein	CRP_HUMAN	2	8.9%	0.016	12.785
Lysosome‐associated membrane glycoprotein 2	LAMP2_HUMAN	2	4.4%	<0.001	9.378
Apolipoprotein (a), Apo (a), Lp (a)	APOA_HUMAN	2	20.1%	0.001	8.028
F‐box only protein 10	FBX10_HUMAN	2	3.3%	0.022	6.577
Serum amyloid A‐2 protein	SAA4_HUMAN	4	31.7%	0.003	4.620
Fatty acid‐binding protein	FABPH_HUMAN	2	14.4%	0.008	4.422
Transgelin	TAGL_HUMAN	19	82.6%	0.013	4.266
Gamma‐glutamylcyclotransferase	GGCT_HUMAN	4	23.4%	0.003	3.728

*Note*: UniProt ID, protein identifier within Universal Protein Resource (UniProt) knowledgebase; Number of unique peptides, peptides on which protein identification was based; % sequence coverage, percentage of sequence of the full‐length protein covered by the unique peptides that were identified.

Abbreviations: EAT, epicardial adipose tissue; HFrEF, heart failure with reduced ejection fraction; HFmrEF, heart failure with mildly reduced ejection fraction; HFpEF, heart failure with preserved ejection fraction.

### 3.3 Analyses of the differential EAT proteome between HFrEF/HFmrEF and HFpEF groups

**FIGURE 2:**
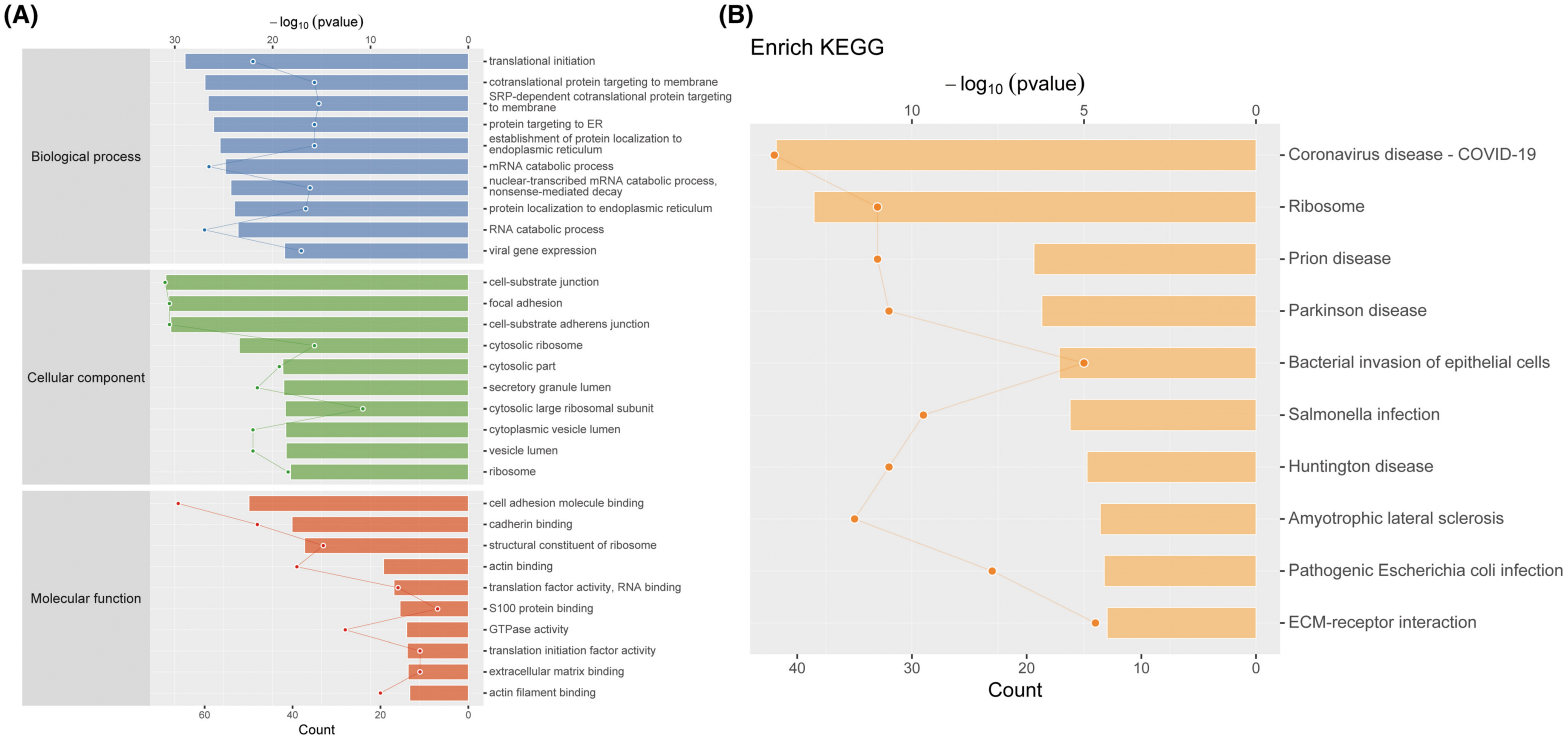
Cellular process mediated by HFrEF/HFmrEF and HFpEF EAT proteins described in network models. (A, B) Gene Ontology and KEGG enrichment analysis of the biological process in the clusters of regulated proteins.

We compared differentially expressed proteins between HFrEF/HFmrEF and HFpEF groups by performing an enrichment analysis of the GO biological process and identified key biological processes and potential pathways that might discriminate HFrEF/HFmrEF from HFpEF. The biological processes significantly represented by the EAT proteome included translational initiation, cotranslational protein targeting to membrane, SRP‐dependent cotranslational protein targeting to membrane, protein targeting to ER, and establishment of protein localization to the endoplasmic reticulum. The enrichment analysis of GO cellular components indicated that most of these proteins were localized in the cell–substrate junction, focal adhesion, cell–substrate adherens junction and cytosolic ribosome. These active factors participated in Coronavirus disease, Ribosome, Prion disease, Parkinson disease, Bacterial invasion of epithelial cells, Salmonella infection, Huntington disease, Amyotrophic lateral sclerosis, Pathogenic *Escherichia coli* infection, ECM–receptor interaction as described in KEGG pathway mapping (Figure 2A,B).

### 3.4 Verified the selected protein

**FIGURE 3:**
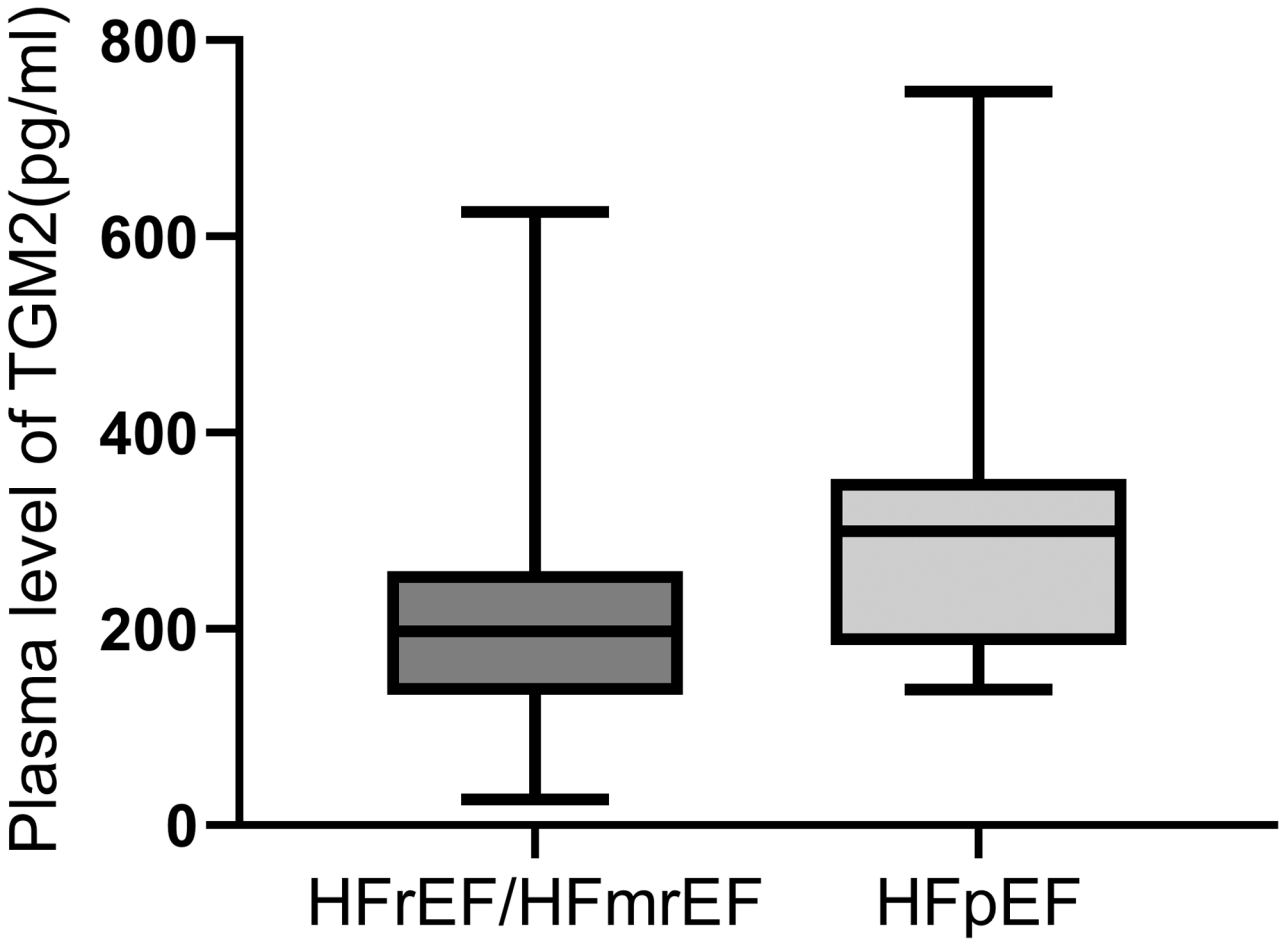
Plasma TGM2 levels were significantly decreased in HFrEF/HFmrEF (*n* = 20) than HFpEF (*n* = 40) (*p* = 0.019). Error bars represent the median with interquartile ranges.

**FIGURE 4:**
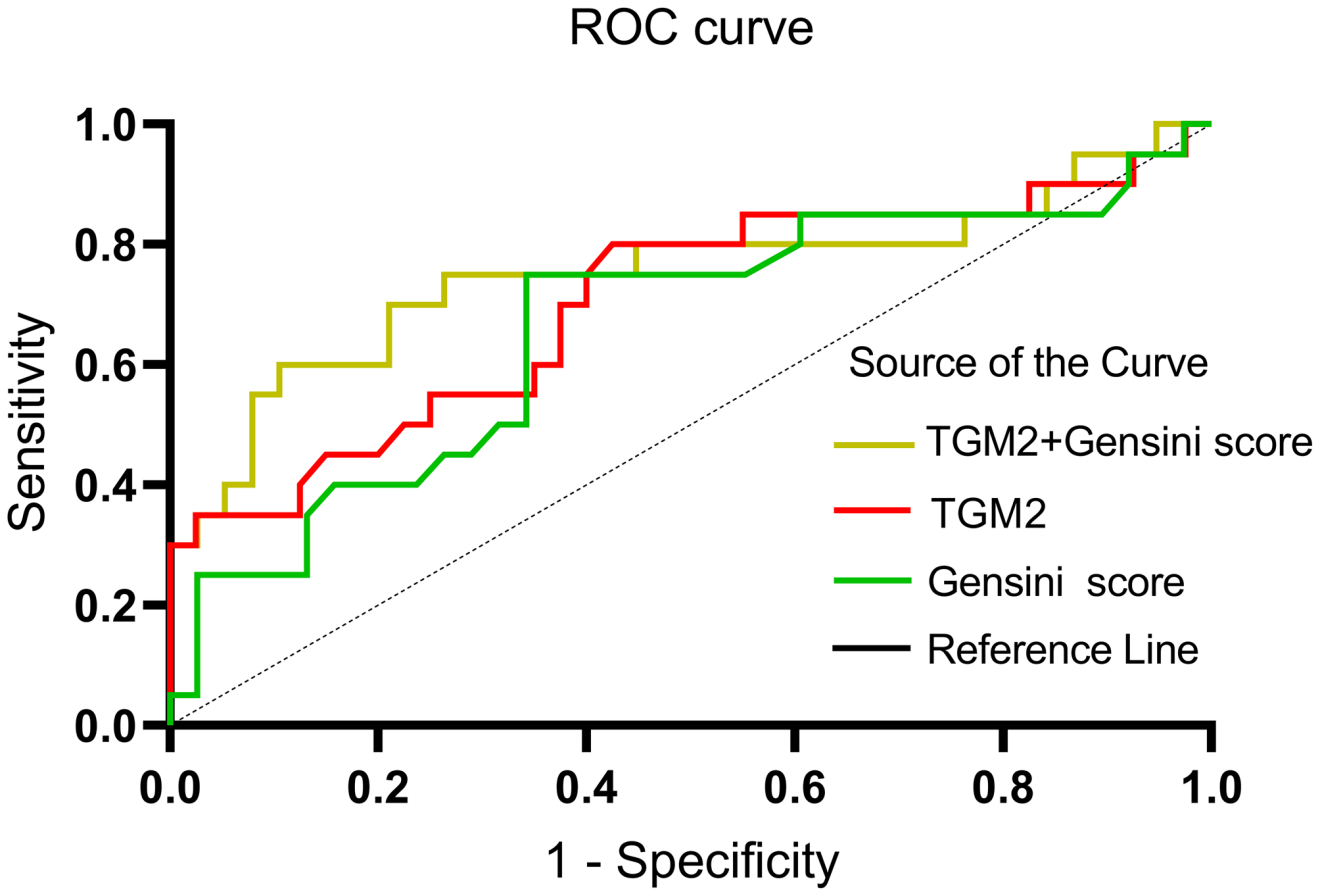
Receiver operating characteristic (ROC) curve for predicting HFrEF/HFmrEF in all patients. AUCs: Combination of TGM2 and Gensini score (yellow line), 0.751; TGM2 (red line), 0.703; Gensini score (green line), 0.662.

Ten candidate EAT proteins which were most significantly up‐ and down‐regulated using label‐free LC–MS/MS were obtained as primary targets for further clinical validation in larger cohorts. As presented in Table 2, TGM2 (Protein‐glutamine gamma‐glutamyltransferase 2) levels were down‐regulated in HFrEF/HFmrEF group, with an HFrEF/HFmrEF to HFpEF ratio of 0.016 and a *p* value less than 0.001. To test the validity of this result, the plasma levels of TGM2 were tested in patients with HFrEF/HFmrEF (*n* = 20) and HFpEF (*n* = 40). The main laboratory data were illustrated in Table 3. Patients in HFrEF/HFmrEF group showed decreased TGM2 levels in plasma compared with HFpEF group [197.8 (133.1, 258.9) vs 299.5 (184.2, 349.6) pg/ml, *p* = 0.019. Table 3, Figure 3] and Gensini scores were significantly higher in HFrEF/HFmrEF group than the HFpEF group (110.1 ± 47.78 vs 84.7 ± 36.53, *p* = 0.028). Further analysis showed that the concentration of TGM2 and Gensini scores were independent risk factors for ejection fraction in HF patients (Table 4). In ROC analysis for the prediction of HFrEF/HFmrEF, the AUC for TGM2 and Gensini scores was 0.703 and 0.662, respectively (95% CI: 0.552, 0.854, *p* = 0.010; 95% CI: 0.507, 0.807, *p* = 0.044), while the AUC for the combination of TGM2 and Gensini scores was 0.751 (95% CI: 0.600, 0.903, *p* = 0.002) (Figure 4 and Table 5). The value of the combination of TGM2 with Gensini scores for predicting HFrEF/HFmrEF was significantly higher than each parameter alone.

**TABLE 3:** Baseline characteristics of the 60 heart failure patients.

	HFrEF/HFmrEF (*n* = 20)	HFpEF (*n* = 40)	*p* value
Age, (years)	68.4 ± 13.4	67.0 ± 12.7	0.699
Male, *n* (%)	14 (70%)	27 (67.5%)	0.544
Gensini scores	110.1 ± 47.78	84.7 ± 36.53	0.028*
SYNTAX score	24.8 ± 6.93	25.5 ± 9.02	0.756
TGM2 (pg/mL)	197.8 (133.1, 258.9)	299.5 (184.2, 349.6)	0.019*
FBG (mmol/L)	7.42 (6.94, 12.02)	8.22 (6.87, 10.35)	0.476
CHO (mmol/L)	4.52 ± 0.90	4.66 ± 0.94	0.600
HDL (mmol/L)	0.88 (0.75, 1.19)	1.04 (0.97, 1.30)	0.069
LDL (mmol/L)	3.07 ± 0.88	3.28 ± 0.88	0.373
TG (mmol/L)	1.33 (0.86, 2.54)	1.19 (0.83, 1.91)	0.466
UA (U/L)	356.4 ± 78.79	347.7 ± 111.69	0.757
Cr (umol/L)	78.1 ± 18.80	77.1 ± 14.38	0.843
CRP (mg/L)	6.4 (5.0, 32.7)	15.6 (6.7, 59.2)	0.392
BNP (pg/L)	297.5 (156.0, 567.5)	352.0 (261.0, 442.0)	0.969
EF (%)	42.5 (39.5, 45.25)	59.0 (55.0, 64.0)	0.018*
LVEDD (mm)	52.3 ± 5.42	45.9 ± 3.59	0.000***

*Note*: Data are mean ± SD or number (%); Gensini score and SYNTAX score, two effective tools used to evaluate the severity of coronary artery disease; TGM2, gene name of Protein‐glutamine gamma‐glutamyltransferase 2. *P < 0.05, ***P < 0.001.

Abbreviations: BNP, brain natriuretic peptide; Cr, creatinine; CRP, C reactive protein; CHO, cholesterol; EF, ejection fraction; FBG, fasting blood glucose; HDL, high‐density lipoprotein; LDL, low‐density lipoprotein; LVEDD, left ventricular end‐diastolic dimension; TG, triglyceride; UA, uric acid.

**TABLE 4:** Logistic regression analysis of independent factors for ejection fraction of heart failure patients.

						95% confidence interval for EXP (B)	
variable	B	SE	Wald	*p* value	Odds ratio	Lower limit	Upper limit
TGM2	−0.007	0.003	5.528	0.033	0.993	0.987	0.999
Gensini score	0.017	0.008	4.895	0.027	1.017	1.002	1.032
constant	−0.615	0.997	0.380	0.537	0.541		

*Note*: Gensini score, effective tool used to evaluate the severity of coronary artery disease; TGM2, gene name of Protein‐glutamine gamma‐glutamyltransferase 2.

**TABLE 5:** Area under the curve of parameters for predicting HFrEF/HFmrEF in all patients.

Variable	AUC	*p* value	95% Confidence interval
			Lower limit Upper limit
TGM2	0.703	0.010	0.552 0.854
Gensini score	0.662	0.044	0.507 0.807
TGM2 + Gensini score	0.751	0.002	0.600 0.903

*Note*: TGM2, gene name of Protein‐glutamine gamma‐glutamyltransferase 2; Gensini score, effective tool used to evaluate the severity of coronary artery disease.

## 4 DISCUSSION

EAT is full of pro‐ and anti‐inflammatory adipokines such as adiponectin, which maintains proper cardiac contractility in physiological conditions.^15^ EAT seems to play an opposite prognostic role in HF patients, as EAT reduction is detrimental in HFrEF/HFmrEF patients, while increased EAT has a positive role in HFpEF patients.^11^ In HFpEF patients, EAT thickness is associated with worse haemodynamic and metabolic outcomes, for example, it is positively correlated with C‐reactive protein, interleukin‐6, uric acid and troponin T levels in HFpEF, which all together give rise to a pro‐inflammatory status in myocardium associated with oxidative stress and fibrosis and are participated in the development of HFpEF. Conversely, in HFrEF/HFmrEF patients, thinner EAT values indicate worsen left ventricular dysfunction, global functional impairment and adverse prognosis.^11^ Furthermore, excessive EAT in HFpEF is associated with the profound hemodynamic disorder at the condition of rest and exercise, including elevated cardiac filling pressures and greater pericardial restraint.^16^ In a type 2 diabetes group, higher EAT thickness is a potential predictor of subclinical cardiac systolic dysfunction.^17^ So far, the molecular mechanisms behind EAT associated with HFrEF/HFmrEF and HFpEF have not been clarified clearly, though some studies indicated that patients with HFrEF expressed significantly lower thermogenic genes in EAT,^18^ suggesting a loss of functional EAT brown‐like features would be deleterious. In the present study, we describe a comprehensive proteomic profile of EAT in both HFpEF and HFrEF/HFmrEF with nano‐LC–MS/MS and demonstrate a group of 599 significantly expressed EAT proteins across EF septum, mainly participating in the regulation of oxidative stress, fatty acid metabolism and collagen metabolic process.

Physiologically, epicardial fat has important functions to maintain cardioprotective effects, including secretion of anti‐inflammatory cytokines, supplement of fatty acids to myocardium during stress situations, and mechanical protection of myocardium and coronary arteries.^19^ Nonetheless, EAT may shift toward a cardiotoxic status under pathological conditions. The oxidative stress and inflammatory factors associated with HFrEF/HFmrEF in our network including unregulated CPM and CRP also took part in macrophage differentiation^20^ and innate immune response^21^ in the previous study. The recruitment of pro‐inflammatory immunocytes and releasing pro‐inflammatory mediators lay a foundation between EAT and cardiac impairment. Also increasingly expressed APOA, FABPH and low levels of DBI in response to reduced EF indicated a disorder of lipid metabolism in EAT, with direct lipotoxic effects on the myocardium.^22^ In addition, HFpEF up‐regulated EAT proteins, participate in collagen fibril organization, such as COL6A6, TNXB, PRTN3 and RAP1A, contributing to increased collagen turnover and severe diastolic dysfunction.^23^ We then constructed protein–protein‐interactive network and validated the candidate protein with a larger cohort. However, future research is warranted to investigate the exact mechanism behind these associations of significantly expressed proteins.

In proteome analysis, TGM2 in EAT is the third down‐regulated protein in HFrEF/HFmrEF patients. Following the validated stage, the peripheral blood concentration of TGM2 is identically decreased in HFrEF/HFmrEF than in HFpEF groups. The consistency of TGM2 down‐regulated in EAT and plasma level indicate that TGM2 as a potential molecule plays an important role in HFrEF/HFmrEF patients. TGM2 is a calcium‐dependent acyltransferase which catalyses the formation of covalent bonds between peptide‐bound glutamine and various other amines, such as gamma‐amino group of peptide‐bound lysine, or mono‐and polyamines, thereby implicating the cross‐linking of proteins such as ACO2, HSPB6, FN1, HMGB1, RAP1GDS1, SPP1^24^, ^25^ and the conjugation of polyamines to proteins.^26^, ^27^ It involves in many biological processes, such as chromatin modification and apoptosis, cellular differentiation, angiogenesis, bone development and wound healing.^28^ Under physiological conditions, the protein cross‐linking activity is inhibited by GTP, but the inhibition is relieved by Ca^2+^ in response to various stresses.^29^ So, when TGM2 is secreted, catalysed cross‐linking of proteins of the extracellular matrix, such as FN1 and SPP1, resulted in the formation of scaffolds.^30^ It plays the role in the development of collagen synthesis and fibrosis in the right ventricle of the heart in a mouse model.^31^ TGM2 is responsible for cross‐linking and stiffening of extracellular matrix and it is involved in the progress of HF.^32^ TGM2 is shown to influence myocardial signalling in contractility.^33^ TGM2 mRNA levels increased in hypertrophied rat ventricles and further increased during the development of HF,^34^ but there is no distinction between whether preserved or reduced ejection fraction in HF. In our study, TGM2 expressions are both down‐regulated in EAT and plasma levels of HFrEF/HFmrEF patients compared with HFpEF patients. Due to the function of apoptosis that keeping the integrity of the dying cells before cleared, down‐expression of TGM2 in HFrEF/HFmrEF patients may prevent the wastage of harmful intracellular components both by catalysing the cross‐linking of cytoskeletal proteins resulting in condensation of the cytoplasm and by mediating cross‐linking proteins of the extracellular matrix resulting in the irreversible formation of scaffolds.^35^ Thereafter, down‐regulated TGM2 indicates worsen outcome in HF with reduced ejection fraction. TGM2 could be a new marker for discriminating HFrEF/HFmrEF from HFpEF patients. Further research on TGM2 will provide new insight for further understanding the mechanism of HFrEF/HFmrEF and the theoretical basis for its treatment.

Nonetheless, we acknowledge the following limitations in this study. First, a relatively small cohort limited further valuable information on the role of candidate EAT proteins. TGM2 should be further verified in a larger‐scale prospective study with a longer duration of follow‐up. Second, The BMI of the HFpEF patients is lower than that of usual HFpEF patients. This may be also related to the small sample size due to the precious source of epicardial tissue. Thirdly, a lack of a control group of patients without HF undergoing EAT proteomic profiling represents another limitation. Fourthly, there is no clear evidence that TGM2 is secreted by EAT and then released into the peripheral circulation. The source still needs further study. Finally, our study is on the discovery phase towards the role of EAT across the EF spectrum. Further basic investigation will be followed.

In conclusion, this current study, for the first time, compares the proteome in EAT between HFpEF and HFrEF/HFmrEF and identifies a comprehensive dimension of potential targets for the mechanism behind the EF spectrum. Our findings suggest EAT is not equal—it has heterogeneous properties that modify cardiovascular and HF risk, offering potential targets for preventive intervention.

## AUTHOR CONTRIBUTIONS

**Qian Gao:** Formal analysis (equal); writing – original draft (equal). **Shan He:** Formal analysis (equal); writing – original draft (equal). **Yuanshu Peng:** Data curation (equal). **Pixiong Su:** Data curation (equal); investigation (equal). Lei Zhao: writing ‐ original draft (equal); revision (equal).

## CONFLICT OF INTEREST STATEMENT

The authors declare that they have no conflict of interest.

## Data Availability

The mass spectrometry proteomics data have been deposited to the Proteome Xchange Consortium via the PRIDE partner repository with the dataset identifier PXD014592 and PXD028158.
